# Cross-presentation of viral antigens in dribbles leads to efficient activation of virus-specific human memory t cells

**DOI:** 10.1186/1479-5876-12-100

**Published:** 2014-04-16

**Authors:** Wei Ye, Yun Xing, Christopher Paustian, Rieneke van de Ven, Tarsem Moudgil, Traci L Hilton, Bernard A Fox, Walter J Urba, Wei Zhao, Hong-Ming Hu

**Affiliations:** 1 Medical School, Southeast University, 87 Dingjiaqiao Street, 210009 Nanjing, Jiangsu, PR China; 2 Cancer Research and Biotherapy Center, the Second Affiliated Hospital, Medical School, Southeast University, 1-1 Zhongfu street, 210003 Nanjing, Jiangsu, PR China; 3 Laboratory of Cancer Immunobiology, Robert W. Franz Cancer Research Center, Earle A. Chiles Research Institute, Providence Cancer Center, Portland, OR, USA; 4 Minigene Pharmacy Laboratory, School of Life Science and Technology, China Pharmaceutical University, 24 Tongjia Xiang, 210009 Nanjing, Jiangsu, PR China; 5 Laboratory of Molecular and Tumor Immunology, Robert W. Franz Cancer Research Center, Earle A. Chiles Research Institute, Providence Cancer Center, Portland, OR, USA; 6 UbiVac, Portland, OR, USA; 7 Department of Molecular Microbiology and Immunology; and Knight Cancer Institute, Oregon Health and Science University, Portland, OR, USA; 8 Cancer Research, Robert W. Franz Cancer Research Center, Earle A. Chiles Research Institute, Providence Cancer Center, Portland, OR, USA

**Keywords:** Autophagosome, Cross-presentation, Immunological monitoring, Immunotherapy biomarker, Viral vaccine, Cancer vaccine, Memory antigen-specific T cells, Proteasome

## Abstract

**Background:**

Autophagy regulates innate and adaptive immune responses to pathogens and tumors. We have reported that autophagosomes derived from tumor cells after proteasome inhibition, DRibbles (Defective ribosomal products in blebs), were excellent sources of antigens for efficient cross priming of tumor-specific CD8^+^ T cells, which mediated regression of established tumors in mice. But the activity of DRibbles in human has not been reported.

**Methods:**

DRibbles or cell lysates derived from HEK293T or UbiLT3 cell lines expressing cytomegalovirus (CMV) pp65 protein or transfected with a plasmid encoding dominant HLA-A2 restricted CMV, Epstein-Barr virus (EBV), and Influenza (Flu) epitopes (CEF) were loaded onto human monocytes or PBMCs and the response of human CMV pp65 or CEF antigen-specific CD4^+^ and CD8^+^ memory T cells was detected by intracellular staining. The effect of cytokines (GM-CSF, IL-4, IL-12, TNF-α, IFN-α and IFN-γ) TLR agonists (Lipopolysaccharide, Polyinosinic-polycytidylic acid (poly(I:C), M52-CpG, R848, TLR2 ligand) and CD40 ligand on the cross-presentation of antigens contained in DRibbles or cell lysates was explored.

**Results:**

In this study we showed that purified monocytes, or human PBMCs, loaded with DRibbles isolated from cells expressing CMV or CEF epitopes, could activate CMV- or CEF-specific memory T cells. DRibbles were significantly more efficient at stimulating CD8^+^ memory T cells compared to cell lysates expressing the same antigenic epitopes. We optimized the conditions for T-cell activation and IFN-γ production following direct loading of DRibbles onto PBMCs. We found that the addition of Poly(I:C), CD40 ligand, and GM-CSF to the PBMCs together with DRibbles significantly increased the level of CD8^+^ T cell responses.

**Conclusions:**

DRibbles containing specific viral antigens are an efficient ex vivo activator of human antigen-specific memory T cells specific for those antigens. This function could be enhanced by combining with Poly(I:C), CD40 ligand, and GM-CSF. This study provides proof-of-concept for applying this strategy to activate memory T cells against other antigens, including tumor-specific T cells ex vivo for immunological monitoring and adoptive immunotherapy, and in vivo as vaccines for patients with cancer.

## Background

Cytotoxic T lymphocytes (CTLs) play critical roles in the clearance of tumor cells and cells infected with intracellular pathogens. Efficient activation of polyvalent and multi-functional antigen-specific CD8^+^ T cells is the Holy Grail for the development of therapeutic vaccines for cancer and chronic infections [1,2]. For tumor-associated antigens (TAAs), the development of a CTL response relies on antigen cross-presentation, a process during which the transformed or over-expressed antigens expressed by tumor cells are captured, processed and cross-presented to CD8^+^ T cells by professional antigen-presenting cells (pAPCs), such as dendritic cells (DCs) [3,4]. The assumption is that most tumor antigens are passively released from healthy or dying tumor cells as intact soluble antigens, peptide fragments complexed with heat shock protein chaperones, or packaged in secretory vesicles in the form of microparticles or exosome nanoparticles. Our previous studies with mouse tumor models identified autophagy as one of the critical pathways regulating antigen packaging and transfer from tumor cells to dendritic cells for cross-presentation to T cells [5,6].

Autophagy is a homeostatic process that involves the sequestration of cytoplasmic components in double-membrane autophagosomes, which subsequently fuse with lysosomes to form autolysosomes, where their cargoes are enzymatically degraded and recycled [7,8]. We have shown that autophagy in tumor cells also plays a critical role in cross-presentation of tumor antigens and identified autophagosomes as antigen carriers for efficient cross-presentation [9,10]. Based on these findings, we developed a novel vaccine strategy to produce DRibbles whose major structure and functional constituents are autophagosomes. Tumor-derived vesicles produced under normal growth conditions contain few ubiquitinated proteins, whereas DRibbles produced from tumor cells treated with inhibitors of proteasomes and lysosomes contain a broad spectrum of cellular antigens, including long-lived proteins, ubiquitinated short-lived proteins (SLiPs), and defective ribosomal products (DRiPs) [9]. DRibbles efficiently cross-prime antigen-specific naïve CD8^+^ and CD4^+^ T cells *in vitro* and *in vivo*; and immunotherapy with DRibbles isolated from a variety of murine tumor cell lines is protective or therapeutic in mice bearing established melanomas, lung carcinomas, breast carcinomas, and sarcomas [10,11]. A pilot Phase I clinical trial using DRibbles derived from autologous tumor cells to vaccinate patients with non-small cell lung cancer has been completed and a randomized multi-center Phase II clinical trial using Allogeneic DRibbles has been initiated (Sanborn, manuscript in preparation). The vaccine employed in the phase II study contains at least 25 putative cancer antigens, including 9 cancer antigens that were “prioritized” by an NCI panel, as well as damage-associated molecular pattern molecules (DAMPs) (Hilton, manuscript in preparation; van de Ven, manuscript in preparation).

Here we report results using DRibbles to activate antigen-specific T cells from humans and to serve as a source of TAA for the immunological monitoring of tumor antigen-specific T cell responses for patients undergoing vaccine trials. Cytomegalovirus (CMV), a ubiquitous herpes virus that infects 50-90% of humans persists in myeloid cells under the control of specific T cells. CTLs play a central role in suppressing CMV reactivation and disease. A relatively high frequency of CMV-specific memory T cells can be found in CMV serum positive donors. The dominant CTL response is directed against the pp65 protein [12]. We devised an ex vivo cross-presentation assay model system, in which DRibbles were made from the pp65-expressing HEK293T embryonic kidney cell line or the non-small cell lung cancer (NSCLC) cell line UbiLT3 and used to stimulate PBMCs isolated from CMV serum positive donors.

In this study, we examined whether pp65 DRibbles derived from either HEK293T or UbiLT3 could activate human pp65-specific memory CD8^+^ T cells and CD4^+^ T cells ex vivo by directly adding DRibbles to PBMCs in culture. Next, we determined the optimal duration for antigen-presenting cells to acquire, process, and cross-present antigens in DRibbles to antigen-specific T cells in PBMCs. Moreover, we tested whether the cross-presentation of DRibbles by human PMBCs could be enhanced by adding “DC licensing factors” such as cytokines and TLR agonists in culture together with DRibbles [13].

## Materials and methods

### Human blood donors and preparation of PBMCs

Leukapheresis products were obtained from healthy volunteers. Written informed consent was obtained from all donors in accordance with the Declaration of Helsinki. Leukapheresis products were cryopreserved in liquid nitrogen. PBMCs were isolated using the density gradient separation method according to the manufactory’s protocol. PBMCs from one of the healthy donors were separated into monocytes and lymphocytes by Elutra apheresis according to the density before cryopreservation. All studies were approved by the institutional review board (IRB) of Providence Portland Medical Center.

### Tumor cell lines and generation of cell lines expressing the dominant antigen pp65 of CMV or CEF composite epitopes

HEK 293 T cells were maintained in Dulbecco’s modified Eagle’s medium (Lonza, 12-604 F) supplemented with 2 mmol/L L-glutamine, 100 units/ml penicillin and 100 ug/ml streptomycin (Invitrogen, 10378), 1 mmol/L sodium pyruvate (Lonza, 13-115E), and 10% fetal bovine serum (FBS, Invitrogen, 16000044). The UbiLT3 cell line was provided by Dr. Traci Hilton (UbiVac, Portland, OR) and maintained in complete medium containing RPMI 1640 (Lonza, 12-702Q), 10% FBS, 2 mmol/L L-glutamine, 100 units/ml penicillin and 100 ug/ml streptomycin and 1 mmol/L sodium pyruvate. The pp65 coding sequence was amplified by PCR from CMV genomic DNA. The HPV E6 E7 coding sequences were obtained from an expression vector (kindly provided by Dr. T. C. Wu at John Hopkins University). The CEF coding sequence was provided by Dr. John R. Webb. All coding sequences were cloned into the p6A-O”PB-IPWS transposon vector. The UbiLT3 pp65 and UbiLT3 GFP control cell line were generated by transfection of UbiLT3 with pp65 plasmid vector or control vector encoding GFP protein and two weeks selection with 2 μg/ml puromycin (Invivogen, ant-pr-1). Expression of pp65 and GFP was confirmed by western blot analysis or GFP fluorescence. These cell populations were used to prepare pp65 DRibbles or control GFP DRibbles. In some experiments, DRibbles were generated from HEK293T cells that were transiently transfected with either a CMV pp65 plasmid, a plasmid encoding dominant HLA-A2 restricted CMV, EBV, and Flu epitopes (CEF) or a plasmid encoding HPV E6E7 (as a negative control).

### Preparation of DRibbles and cell lysate

Autophagosome-enriched DRibbles were prepared as described previously [9]. Briefly, cells were treated with Bortezomib (200 nmol/L, Millennium) and ammonium chloride (10 ~ 20 mmol/L, Sigma Aldrich, A9434) for 24 to 48 hours, cells and cellular debris were pelleted by centrifugation at 300 × *g* for 7 minutes. DRibbles were dislodged from cells or clumps of cell debris by rigorous pipetting. The suspension was then centrifuged at 7500 × *g* to pellet the DRibbles and discard supernatant containing nanovesicles and exosomes. Total cell lysates were produced by three freeze thaw cycles: freezing in a dry-ice enthanol bath followed by thawing in a 37°C water bath. The total amount of protein in DRibbles and lysates was quantified using a BCA protein assay Kit with the assay performed according to the manufacturer’s protocol (Thermo scientific, 23228).

### Resting of PBMCs and determine the optimal condition for DRibbles stimulation of memory T cells ex vivo

For experiments with monocytes and lymphocytes isolated by leukapheresis, monocytes were first thawed, rested in complete medium containing RPMI 1640, 10% FBS, 2 mmol/L L-glutamine, 100units/ml penicillin, 100ug/ml streptomycin and 1 mmol/L sodium pyruvate (RP10) for 12 hours and seeded into a 96-well round-bottomed plate at 1 × 10^5^ cells per well. DRibbles were added to specified wells at indicated concentration and lymphocytes were thawed and promptly added at 5 × 10^5^ cells per well. After 12 h stimulation, brefeldin A (10ug/ml, sigma-aldrich, B7651) was added to the culture for another 6 hours before they were harvested and stained for flow cytometric analysis.

Frozen PBMCs were thawed and rested in RP10 medium or the medium containing complete medium and X-VIVO™ 15 (Lonza, 04-418Q) at the ratio of 1:1 (RX15) for 6 ~ 12 hours. Next, rested cells were seeded into a 96-well round-bottomed plate at 0.5 ~ 1 millions cells per well and DRibbles, cell lysate or protein were added at indicated amount. After 12 h of incubation with DRibbles, brefeldin A (10 ug/ml) was added to the culture and kept in culture for another 12 hours.

To identify the optimal culture condition for DRibbles-induced T-cell activation, rested PBMCs were stimulated by DRibbles in the presence of different DC licensing factors. These factors included recombinant GM-CSF (40 ng/ml, immunex), IL-4 (10 ng/ml, peprotech, 200-04) and IFN-α-2b (1000 U/ml, intron A). Some factors were added at the same time with antigens including Lipopolysaccharide (100 ng/ml, immunechem), Poly(I:C) (25 ug/ml, invivogen, tlrl-piclv), M52-CpG (30 ug/ml, Sigma), R848 (10 ug/ml, invivogen, tlrl-r848), TLR2 ligand (300 ng/ml, invivogen, tlrl-pms), CD40 ligand (1 ug/ml, NIH repository, D759060X), IL-1α(300 ng/ml, NIH repository, Zlva0903), TNF-α (1000 U/ml, Cetus, 94608), and IFN - γ (100 ng/ml, peprotech, 30002).

### Antibodies and western blot analysis

Cell lysates or DRibbles were mixed with 4× NuPAGE LDS sample buffer. Samples were sonicated and boiled in 85°C bath for 5 minutes. Samples (10 ug protein in 10ul for each) were resolved by 4% to 20% SDS-PAGE and transferred to PVDF membranes. Memberanes were blocked 1 h in PBS-Tween 0.1% containing 5% skim milk powder and incubated overnight with anti-ubiquitin (1:2,000, 5379, Sigma) and anti-CMV pp65 (1:1,000, 52401, Santa cruz) at 4°C overnight. Next day membranes were incubated with HRP-labeled goat-anti-mouse (1:5,000, 31430, Thermo) and goat-anti-rabbit (1:20,000, 31460, Thermo) secondary antibodies for 1 h and developed with chemiluminescent reagents (1705070, Bio-Rad).

### Analysis of antigen-specific T cells by intracellular IFN-γ staining and flow cytometry analysis

After stimulation, PBMCs were collected into FACS tubes and stained with live/dead fixable dead cell staining (Invitrogen, L34957) and stained with antibodies against CD3 (peridinin-chlorophyll proteins, Invitrogen, MHCD0331), CD4 (Alexa Fluor® 488, Invitrogen, MHCD0420), CD8 (Phycoerythrin, Invitrogen, MHCD0804), and IFN-γ (APC, Invitrogen, MHCIFG05) and analyzed by a custom-build LSRII flow cytometer (BD bioscience). Data were collected with BD FACSDiva software and analyzed with Treestar Flowjo software.

### Statistical analysis

Non-paired or paired *t* test were performed using Prism (GraphPad Software). *P* < 0.05 with two tails represented difference was statistically significant.

## Results

### Optimization of cross-presentation and intracellular IFN-γ staining assay for the DRibble-induced human T-cell responses

**Figure 1:**
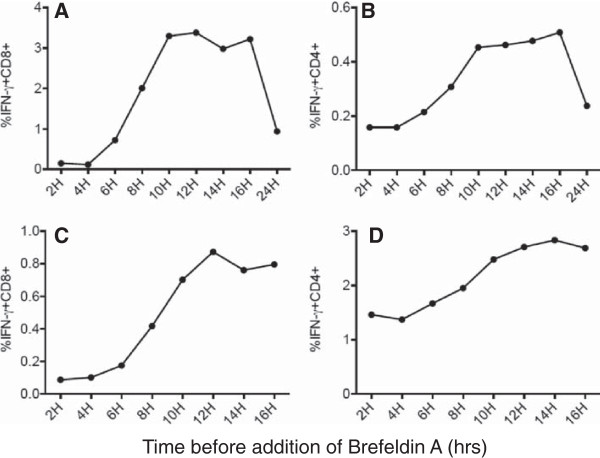
**The kinetics of CD4 and CD8 T-cell activation following exposure to DRibble-pulsed APC. (A,B)** PBMCs were separated into monocytes and lymphocytes by Elutra apheresis. Monocytes were pulsed with CEF DRibbles for 6 hours. After that time autologous T cells were added into wells at a ratio of 5:1 (T cells:monocytes). Brefeldin A was added to the culture after the time periods specified in the figure. All cells were harvested at the same time and prepared for flow cytometry. **(C,D)** PBMCs (monocytes and T cells) were cultured with pp65 DRibbles and then Brefeldin A was added to the culture after the time periods specified in the figure. Cells were harvested at the same time and prepared for flow cytometry.

We previously reported that tumor-derived DRibbles when pulsed onto bone marrow-derived DC were highly efficient carriers of antigen for cross-presentation to naïve CD8^+^ T cells in mice [9]. To investigate whether DRibbles were also efficient activators of antigen-specific T cells in humans, elutriated monocytes were used as antigen-presenting cells and exposed to DRibbles made from cells expressing viral antigens. Matched Lymphocytes that were autologous to the elutriated monocytes and with pre-existing memory T cells specific for viral antigens were used as the responder cells. DRibbles were prepared from HEK293T cells that had been transiently transfected to express the major T-cell epitopes of CMV, EBV, and influenza virus (termed as CEF) or E6E7 fusion proteins of HPV as the negative control (the donor was HPV seronegative) [14]. To measure antigen cross-presentation, we used the intracellular staining (ICS) assay and flow cytometric analysis to determine the frequency of antigen-specific T cells by measuring the production of IFN-γ. Initial experiments sought to determine the conditions for the processing and presentation of DRibbles that achieved optimal activation of antigen-specific T-cell responses. HEK 293 T CEF DRibbles were loaded onto monocytes for 6 hours, after which T cells were added into the culture. Brefeldin A was added at different time points after T-cell addition to determine the duration of T-cell stimulation required for optimal production and retention of intracellular IFN-γ. Subsequently, all cells were harvested at the same time and the ICS was performed by flow cytometry to calculate the percentage of IFN-γ^+^ T cells. As the culture period without brefeldin A was extended from 2 hours to 10 hours both the CD8^+^ and CD4^+^ T-cell responses increased with a plateau between 10 hours to 16 hours (Figure 1A,B). This was followed by a significant decline at 24 h.

Next, we determined the optimal timing for the addition of brefeldin A after direct loading of DRibbles onto PBMC without monocyte isolation and the preloading of DRibbles specified above. Analysis of intracellular IFN-γ staining revealed that the highest T-cell responses were detected when brefeldin A was added to culture 12 hours after stimulation (Figure 1C,D). Collectively, these data show that when cryopreserved PBMC are assayed, it takes about 12 hours for DRibbles to be cross-presented and to activate T cells before adding brefeldin A and assaying IFN-γ.

### Human monocytes or PBMCs “loaded” with DRibbles efficiently stimulate memory CD8^+^ T cells and CD4^+^ T cells

**Figure 2:**
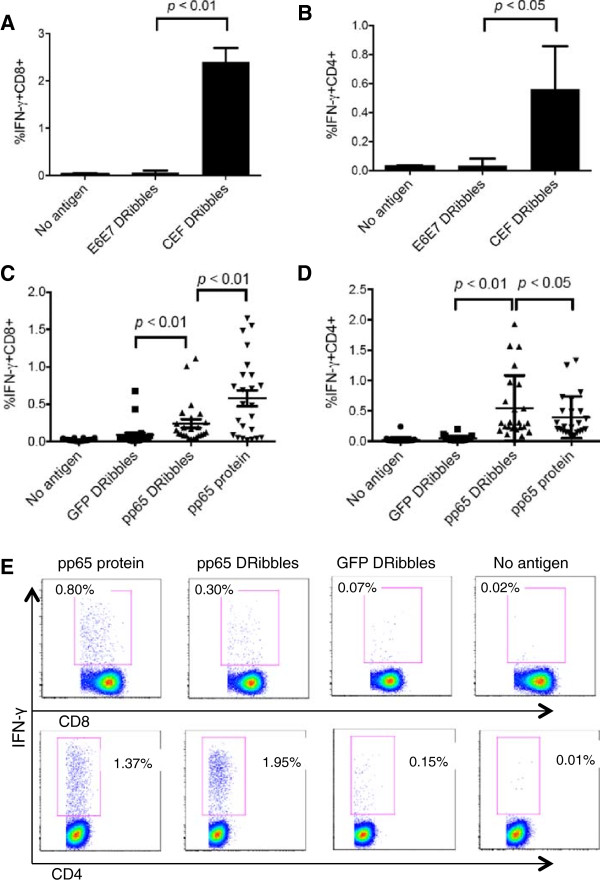
**DRibbles were efficient antigen carriers for the activation of human CD8**^**+ **^**T cells and CD4**^**+ **^**T cells.** PBMCs were separated according to their density into monocytes and lymphocytes by Elutra apheresis. DRibbles were collected from HEK 293 T cells that expressed E6E7 protein or CEF protein. Monocytes were loaded with 25ug/ml CEF DRibbles or 25ug/ml control E6E7 DRibbles. After 6 hours, lymphocytes were added. Activation of T cells was assessed by determining the percentage of IFN-γ^+^CD8^+^ cells **(A)** and IFN-γ^+^CD4^+^ cells **(B)** detected by ICS. The mean ± SEM of 3 separate experiments from the same donor are shown. CD8^+^**(C)** and CD4^+^ T cell responses **(D)** against UbiLT3 pp65 DRibbles or control UbiLT3 GFP DRibbles and CMV pp65 protein were analyzed in frozen-thawed PBMCs from 24 subjects. **(E)** shows the representative dot plot from one of the donors.

With the assay optimized we set out to evaluate the capacity of DRibbles to characterize CEF and particularly CMV pp65 immunity across a population of donors. In these studies monocytes loaded with CEF-DRibbles stimulated more CD8 T cells than control E6E7-DRibbles (2.4% vs 0.06%; *p* < 0.01; Figure 2A) and CD4^+^ T-cells (0.56% vs 0.03%; *p* < 0.05; Figure 2B). Stimulation with E6E7-DRibbles did not increase the response beyond the frequency observed in the no antigen groups. These results showed that, when loaded onto monocytes, HEK 293 T derived DRibbles, cross-presented viral antigens to both antigen-specific CD8^+^ T cells and CD4^+^ T cells response. There was no apparent non-specific stimulation of memory T cells by DRibbles derived from HEK 293 T cells lacking the CEF antigenic peptides.

We also used DRibbles derived from the UbiLT3 cell line, which was derived from a patient with NSCLC. A line of UbiLT3 that expressed the pp65 protein of CMV was generated and used to prepare DRibbles as a source of CMV pp65 antigens to stimulate PBMCs from 24 donors. The percentage of IFN-γ^+^CD8^+^ T cells and IFN-γ^+^CD4^+^ T cells were calculated following intracellular staining protocol described above. The mean percentage of IFN-γ^+^CD8^+^ T cells was 0.24% for the UbiLT3 pp65 DRibbles-stimulated group compared to 0.08% in the paired group of T cells treated with control UbiLT3 GFP DRibbles (*p* < 0.01; Figure 2C,E). The mean percentage of CD4^+^ T-cells that produced IFN-γ upon pp65 DRibbles stimulation was 0.54%, vs 0.04% following stimulation with control DRibbles (*p* < 0.01; Figure 2D,E). Compared to purified recombinant CMV pp65 protein, UbiLT3 pp65 DRibbles were better stimulators of memory CD4^+^ T cells (0.54% vs 0.39%, *p* < 0.05, Figure 2D,E) but less stimulatory to memory CD8^+^ T cells (0.24% vs 0.58%, *p* < 0.01, Figure 2C,E). These data show that DRibbles from HEK293T cells and UbiLT3 cells are potent immunogens for antigen-specific activation of CD8^+^ T cells and CD4^+^ T cells when loaded onto elutriated monocytes or added directly to human PBMCs.

### Poly (I:C) enhances the cross-presentation of DRibble antigens by PBMCs

**Figure 3:**
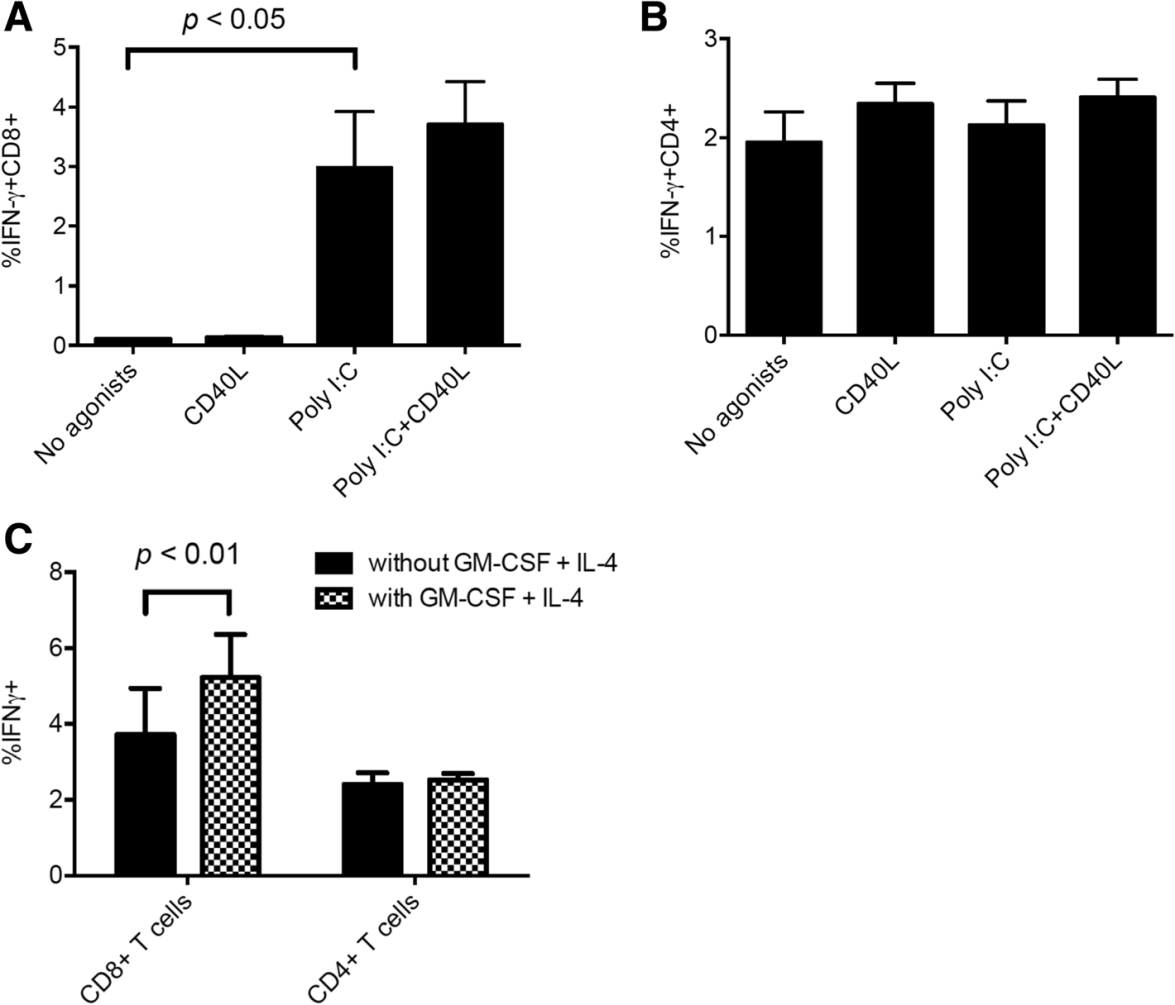
**CD8**^**+ **^**T cell response can be enhanced by adding Poly (I:C) and GM CSF/IL-4 to DRibble-pulsed PBMCs.** Poly (I:C) and CD40L were added with UbiLT3 pp65 DRibbles (25ug/ml) to rested PBMCs. The mean ± SEM of 3 separate experiments are shown. **(A)** represents the percentage of IFN-γ producing CD8^+^ T cell and **(B)** represents the CD4^+^ T cell response. **(C)** PBMCs were cultured with or without GM-CSF and IL-4 for 12 hours, then Poly (I:C) and CD40L were added to PBMCs with UbiLT3 pp65 DRibbles (25ug/ml). The mean ± SEM of 3 separate experiments are shown.

We recently demonstrated that DRibbles express CLEC9A ligand and were efficiently cross-presented by mouse CLEC9A^+^ DC [9]. In humans, CLEC9A expression is restricted to the subset of BDCA3(CD141)^+^ DC. Because of its superior ability to cross present cell-associated antigens, investigators have focused on this DC subset. It is generally accepted that human BDCA3 (CD141)^+^ DC are the human counterpart of mouse CD8α^+^ cDCs which can efficiently capture dead or dying cells, process, and cross-present exogenous antigens for the induction of protective CD8^+^ cytotoxic T cell responses [15,16]. Several studies have revealed that human CD141^+^ mDCs expressing high levels of TLR3 could cross-present exogenous antigens efficiently when the TLR3 agonist, poly (I:C), was added to the assay [17,18]. Thus we tested whether the addition of poly (I:C) to PBMCs with UbiLT3 pp65 DRibbles would further increase the percentage of pp65-specific IFN-γ^+^ T cells. Figure 3A shows that the addition of poly (I:C) to DRibbles significantly enhanced the percentage of IFN-γ producing CD8^+^ T cells as compared to DRibbles without poly (I:C) (2.99% vs 0.10%, *P* < 0.05). The combination of CD40L and Poly (I:C) did not further increase the CD8^+^ T cell response and CD40L alone did not significantly increase the percentage of IFN-γ-secreting CD8^+^ T cells (Figure 3A). Neither the addition of poly (I:C) nor CD40L affected the CD4^+^ T cell responses (1.96% vs 2.13%, *P* > 0.05, Figure 3B). We found that under these inflammatory conditions the pp65 specific CD8+ and CD4+ T cell responses stimulated by DRibbles is still dependent on antigen presenting cells (Additional file 1: Figure S1).

The addition of other TLR agonists showed that Poly (I:C), CpG, and LPS could enhance the cross-presentation of UbiLT3 pp65 DRibbles, whereas R848 (TLR7/8 agonist) and TLR2 agonist had no effect on the CD8^+^ T-cell response (Additional file 2: Figure S2A). The pp65 specific CD4^+^ T cell response was not affected by any of the TLR agonist tested (Additional file 2: Figure S2B).

### GM-CSF further enhances DRibble-induced CD8^+^ T-cell responses

Martinuzzi and colleagues reported that GM-CSF/IL-4 and maturation factors induced accelerated cocultured DCs from unfractionated PBMCs and enhanced Ag-specific T-cell stimulation [19]. Thus we investigated whether the addition of GM-CSF/IL-4 would further enhance the Ag-specific T-cell stimulation by DRibbles. PBMCs were cultured in media supplemented with GM-CSF and IL-4 for 12 hours; poly (I:C), CD40L and UbiLT3 pp65 DRibbles were added into the culture system, brefeldin A was added and the percentage of IFN-γ^+^ T cells was measured by flow cytometry. The addition of GM-CSF and IL-4 increased the percentage of Ag-specific CD8^+^ T cells from an average of 3.71% to 5.22%, *p* < 0.01 (Figure 3C), but did not increase the Ag-specific CD4^+^ T cell response (2.53% vs 2.41%, *p* > 0.05, Figure 3C). GM-CSF, but not IL-4, was required for the increase in DRibbles stimulated pp65-specific responses; no significant difference was found when IL-4 was omitted from the culture (Additional file 3: Figure S3A,B). In addition, we also tested type I interferon (IFN-α-2b), type II interferon (IFN-γ), IL-1α, TNF-α, and they did not have a significant effect (Additional file 3: Figure S3C,D,E,F). Thus, GM-CSF was included for the following experiments.

### Proteasome inhibition positively affects the ability of DRibbles to induce pp65-specific CD8^+^ T cell response

**Figure 4:**
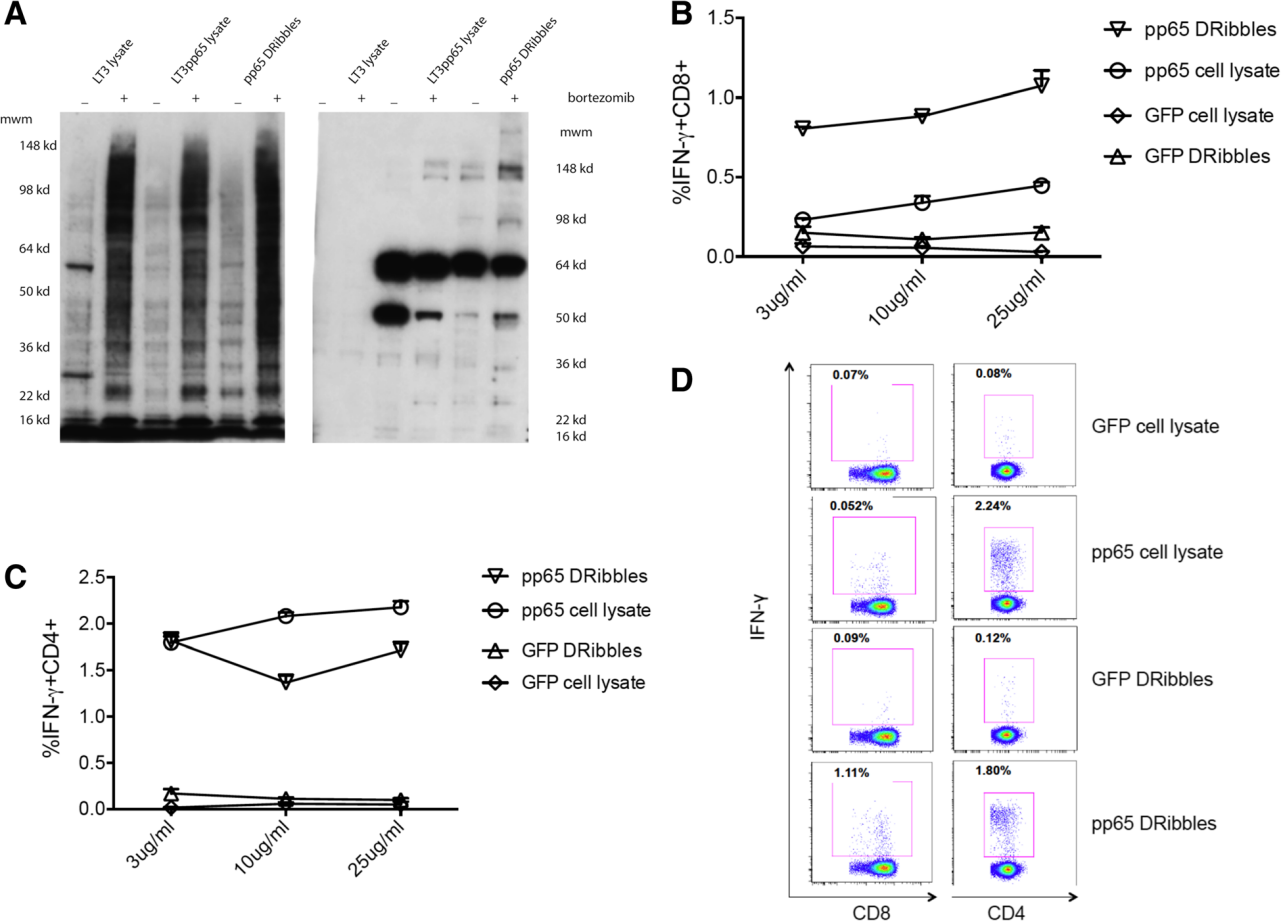
**Antigen-specific CD8**^**+ **^**and CD4**^**+ **^**T cells response following stimulation by DRibbles or cell lysates. (A)** The ubiquitinated proteins and pp65 proteins contained in the cell lysate and DRibbles were examined by western blot. Lysates and DRibbles extracted from cell lines treated with bortezomib contained more ubiquitinated proteins than those from non-treated cells (left). The pp65 protein was detected in the lysates and DRibbles extracted from the UbiLT3 pp65 cell line. The pp65 DRibbles collected from the bortezomib-treated UbiLT3 pp65 cell line contained more 148Kd pp65 protein compared with the other groups (right). Data are representative of 3 independent experiments. **(B,C)** PBMCs were stimulated by DRibbles and cell lysates at the following doses: 3ug/ml, 10ug/ml and 25ug/ml. The percentage of IFN-γ^+^CD8^+^**(B)** and IFN-γ^+^CD4^+^ cells **(C)** were calculated by flow cytometry. Percentages of IFN-γ^+^ T cells are shown as mean ± SEM. Data are representative of results from 3 independent experiments. **(D)** Dot plots at the antigen dose of 25ug/ml.

We previously demonstrated that proteasome inhibition resulted in accumulation of ubiquitinated proteins, which were shunted into autophagosomes in mouse tumor models and following vaccination improved cross-presentation of short-lived proteins [5,9,11]. Here, we sought to extend these observations to the human system. We prepared lysates and DRibbles from untreated UbiLT3 cells or cells in which proteasome function was inhibited by bortezomib treatment and performed western blots with anti-ubiquitin and anti-pp65 antibodies (Figure 4A). We found that ubiquitinated proteins were greatly enriched in cell lysates after bortezomib treatment and similar amounts of pp65 protein were found in lysates and DRibbles regardless of bortezomib treatment. The higher molecular weight bands recognized by anti-pp65 antibody in DRibbles likely represent accumulation of ubiquitinated pp65 proteins in DRibbles following proteasome inhibition.

The UbiLT3 pp65 cell line and control UbiLT3 cell line were either untreated or treated with bortezomib before DRibbles were isolated and added to PBMCs to activate antigen-specific T cells under optimized conditions. Our results showed that DRibbles isolated from bortezomib-treated tumor cells induced more antigen-specific CD8^+^ T cells than DRibbles from untreated tumor cells (Additional file 4: Figure S4). It is worth noting that following proteasome inhibition stimulation with DRibbles increased CD8^+^ T cell responses, however, proteasome inhibition had no effect on stimulation by cell lysates. Importantly, when compared at equivalent doses, UbiLT3 pp65 DRibbles stimulated antigen-specific CD8^+^ T cells more effectively than UbiLT3 pp65 cell lysates (*P* < 0.05, Figure 4B). Surprisingly, DRibbles were either equivalent (at low dose) or slightly less effective (at high doses) than total cell lysates at stimulating CD4^+^ T-cell responses (*P* < 0.05, Figure 4C,D). Thus, DRibbles as a pp65 antigen source has a greater ability to cross-prime CD8^+^ T cells than tumor lysates. The increased CD8^+^ T cell activation seemed to correlate well with enrichment of ubiquitinated pp65 proteins in DRibbles, but not lysates.

## Discussion

In contrast to the recent clinical success of adoptive T-cell immunotherapy and antibodies that interfere with T cell checkpoints, which prove the therapeutic value of T cells, active-specific immunotherapy with cancer vaccines has been disappointing and much less effective. Many reasons, including poor immunogenicity, immune evasion and immune suppression could explain this failure [20]. Approaches to induce a robust antigen-specific CTL response against multiple cancer-associated antigens, which appears to be required for the clearance of tumor cells by the immune system has become a major focus for the development of therapeutic cancer vaccines. CTL recognition of tumor cells, or normal cells infected with viruses, is via peptides, that are presented by MHC I complexes. The dominant peptides contained in MHC I on the cell surface are thought to be derived from short-lived proteins or defective ribosomal products (DRiPs) [9,10]. To increase the cross-priming of tumor-specific CTL, we developed a novel vaccine based on tumor-derived autophagosomes (DRibbles), in which short-lived proteins and DRiPs could be highly enriched after proteasome and lysosome function were inhibited. DRibbles induce efficient cross-presentation, activate naïve CD8^+^ T cells *in vivo* and *in vitro,* and induce cross-protective and therapeutic antitumor immune responses against multiple, antigen distinctive, sarcomas [11] and mammary carcinomas (Twitty C et all manuscript in preparation, Li Y et al manuscript in preparation).

In this study, we sought to determine whether DRibbles could be used to cross-present viral antigens to human T cells. We showed that DRibbles derived from either the HEK293T cell line or UbiLT3 cell line expressing the CMV pp65 antigen accumulated both native and ubiquitinated pp65 protein and efficiently activated pp65-specific human CD4^+^ and CD8^+^ T cells when loaded onto monocytes or directly to unfractionated PBMCs *in vitro*. For the optimal activation of antigen-specific T cell responses in PBMCs, it requires at least 10 hours of processing and presentation of DRibbles by APCs. Moreover, we found that cross-presentation efficiency was significantly enhanced by adding GM-CSF + IL-4, CD40L, and the TLR3 agonist (poly(I:C)) to the culture with DRibbles.

In this study, we used artificial CEF and CMV pp65 as model antigens to investigate whether they could be cross-presented and stimulate T cell responses from pre-existing human memory T cells. CEF is a synthetic antigen comprising 32 MHC class I restricted epitopes from CMV, EB and influenza virus, which are recognized by human donor viral antigen-specific CD8^+^ T cells [14]. Initially, we loaded conventional monocyte-derived DCs with CEF-DRibbles to activate T cell responses, but found that freshly isolated monocytes could also cross-present DRibble antigens to CD8^+^ T cells very efficiently without *ex vivo* culture. The monocytes were isolated from PBMCs by elutriation, but it is very likely that blood DCs were also present and participated in the cross-presentation of antigens from DRibbles. Recently, it has been reported that the CLEC9A^+^BDCA3^+^ DC subset in human PBMCs and tissues can efficiently cross-present cell death-associated antigens [15,21,22]. We can only speculate that the efficient cross-presentation of DRibble antigens was mediated by these DCs, a notion that is being investigated in the lab currently (Hilton, manuscript in preparation; van de Ven, manuscript in preparation).

Based on these initial results, we added DRibbles directly to PBMCs to investigate their ability to activate pre-existing memory CD8^+^ and CD4^+^ T cells response without prior APC isolation and antigen loading. We chose DRibbles derived from the UbiLT3 and HEK293T cell lines that expressed the CMV pp65 antigen, because pp65 is a dominant antigen recognized by both memory CD8^+^ and CD4^+^ T cells in humans with a history of CMV infection [12]. In addition to their ability to stimulate CD8^+^ T cells, DRibbles efficiently induced CMV-specific human CD4^+^ T cells with a similar or slightly higher efficiency than purified pp65 protein. Thus DRibbles can also be presented and cross-presented by APCs in humans and stimulate both CD4 and CD8 T cells, respectively.

In this study, intracellular IFN-γ production was the surrogate measure for effective cross-presentation. The process from cross-presentation of DRibbles by APCs to IFN-γ production by memory T cells is lengthy and complex one; it comprises DRibbles uptake, protein degradation, peptide transport and loading, and finally presentation to T cells. Thus, it is not surprising that it took more than 10 hours for DRibbles to induce efficient CD8^+^ and CD4^+^ T cell responses, whereas it only takes 2-6 hours for peptide antigen pulsed APCs to stimulate IFN-γ production [23]. This information will allow the further optimization of assays for monitoring T cell responses of patients on clinical trials.

Three subsets of human peripheral blood DCs have been described: pDCs; BDCA1^+^ cDCs; BDCA3^+^CELC9A^+^ cDCs [24]. Efficient cross-presentation generally requires DC activation signals. These signals are often triggered by engagement of their TLRs [13]. Prior reports have shown that APCs respond to TLR ligands in accordance with their TLR expression pattern. When pDCs, which express TLR7 and TLR9, are stimulated with either CpG or R848, they exhibit a dramatic and rapid up-regulation of the co-stimulatory molecules CD40, CD80, CD86, CD83, as well as MHC class II molecules, and production of IFN-α [13,25,26]. BDCA1^+^ DCs express a broad spectrum of TLRs, and can respond to poly (I:C), LPS, and R848 [27]. BDCA3^+^CLEC9A^+^ cDCs, a newly described DC subset that expresses XCR1 and TLR3, have the highest capacity to cross present cell-associated antigens [28]. This DC subset is analogous to the mouse CD8α^+^ DCs. Consistent with their high level of TLR3 expression, studies have shown that stimulation with poly (I:C) leads to secretion of large amounts of IL-12 p70, IFN-γ, and most importantly enhanced ability to cross present antigen [22,27].

We recently showed that DRibbles were recognized by CLEC9A and that cross-presentation of DRibbles by mouse DCs was decreased if DRibbles were preincubated with soluble CLEC9A protein as a decoy receptor [9]. Thus, we posit that DRibbles can be most efficiently cross-presented by human blood BDCA3^+^CLEC9A^+^ DCs. Our preliminary cell sorting studies also support this notion. We investigated the effect of different TLR agonists on the cross-presentation ability of PBMCs. Our data showed that poly (I:C), CpG, and LPS can enhance the cross presentation of DRibbles, particularly poly (I:C). The CD8^+^ T cell response to DRibbles appeared to be enhanced when recombinant CD40L was added to the culture. These observations are consistent with recent publications showing that optimal cross-presentation by blood BDCA3^+^CLEC9A^+^ DCs requires TLR3 signaling and a licensing factor, such as CD40L [18,19].

Martinuzzi E et al, found that accelerated cocultured DC (acDC), produced by culturing PBMCs with GM-CSF and IL-4 for 24 h and then adding maturation stimuli for an extra 24 h, could enhance the expansion of antigen-specific T cells and that acDC-amplified T-cell responses were antigen specific [19]. Similarly, we showed that PBMCs could efficiently stimulate T-cell responses when they were cultured with GM-CSF plus IL-4 for 12 h, followed by DRibbles and Poly (I:C)/CD40L for an extra 24 h. However, minor differences in the effect of cytokines were observed. For example, adding IL-4 was not needed for efficient cross-presentation and adding CD40L did not have a significant effect. In addition, the cross-presentation of DRibbles can be decreased dramatically when exogenous IFN-α was added to the culture together with DRibbles. Thus, for efficient cross-presentation of DRibbles, only GM-CSF is needed and sufficient for the rapid functional differentiation of blood DCs when in the presence of DRibbles. DRibbles contain many endogenous agonists for multiple TLRs expressed by monocytes and DCs, which could induce rapid differentiation of monocytes or other DC precursors in human blood when combined with GM-CSF. After that we compared the ability of poly (I:C) plus different cytokines to enhance CD8^+^ T cells response in the background of GM-CSF. We conclude that GM-CSF can sustain the survival and function of APCs during both recovery of PBMCs from cryopreservation and antigen loading and presentation, while adding poly (I:C) plus CD40L at the same time of antigen loading appeared to be the optimal condition that should be adopted. The negative effect of adding exogenous IFN-α on the cross presentation of DRibbles needs further investigation. IFN-α was shown to be the critical cytokine for cross-priming of antitumor immune response in mice [29,30]. However, adding IFN-α to monocytes together with GM-CSF for three days of culture prior to DRibbles loading was found to be beneficial for functional differentiation of DCs, consistent with published work showing that blood monocyte can be induced to differentiate into DCs stimulated by GM-CSF plus IFN-α [31].

The CMV pp65, nonessential protein for virus replication, is the most abundant tegument protein and major constituent of extracellular viruses. Although, it is not known as a short-lived protein, treatment of pp65 expressing cells with proteasome inhibitor enhanced its cross presentation efficiency when either whole cell or DRibbles were used to induce the activation of antigen specific CD8^+^ T cells. However, proteasome inhibitor treatment did not have any effect when cell lysate antigen was used. Furthermore, proteasome inhibition seems to have much less effect on CD4^+^ T cell responses to DRibbles or cell lysates. We speculate that stable long-lived pp65 proteins are major antigens that are presented to CD4^+^ T cells, whereas ubiquitinated pp65 proteins are major antigens presented to CD8^+^ T cells as it appeared that more ubiquitinated pp65 proteins can be enriched in bortezomib treated DRibbles and levels of ubiquitinated pp65 rather than levels of natively folded pp65 correlated with increased CD8^+^ T cells activation after proteasome inhibition. In murine models, we showed that the cross-presentation of short-lived antigens can be enhanced by inducing cell autophagy with a proteasome inhibitor and blocking p62-dependent accumulation of DRiPs in DRibbles diminished the cross-presentation [5].

Inactivated whole tumor cells or tumor cell lysates are commonly used in traditional vaccine strategy for cancer immunotherapy [32]. One major advantage of our novel DRibbles vaccine is the enrichment of short-lived proteins or ubiquitinated DRiPs of misfolded proteins. The majority of these SLiPs and DRiPs are not expected to be contained in vaccines of inactivated whole tumor cells or tumor cell lysates due to rapid degradation. Thus, whole cell or cell lysate vaccines will not efficiently cross-present these tumor/tumor-associated antigens derived from SLiPs and DRiPs. Because SLiPs and DRiPs are not normally cross-presented tumor-bearing hosts are unlikely to be tolerant to these antigens. Thus, tumor-associated antigens contained in SLiPs and DRiPs represent a repertoire of neo-antigens against which the host should be able to generate a strong immune response [6]. Our current work suggests that combinations with other adjuvants may increase the immune response directed against the DRibble vaccine. The ongoing multicenter phase II clinical trial will evaluate the ability of two adjuvants, GM-CSF and imiquimod, to further augment immune responses against tumor-associated antigens.

## Conclusions

Our study highlights the ability of DRibbles to activate memory antigen-specific CD8^+^ and CD4^+^ T cells in human peripheral blood. The results demonstrate that DRibbles can efficiently cross-present antigens to human memory T cells. Coupled with our experience in preclinical cancer models, these data suggest that DRibbles may be an excellent vehicle for priming and boosting anti-cancer immune responses in patients as well as for monitoring anti-cancer immune responses. We are currently testing this strategy in a multicenter randomized phase II clinical trial for patients with definitively treated stage IIIA/B non-small cell lung cancer.

## Abbreviations

CTL: Cytotoxic T lymphocyte; TAA: Tumor-associated antigens; pAPC: Professional antigen presenting cell; DC: Dendritic cell; DRiPs: Defective ribosomal products; CMV: Cytomegalovirus; NSCLC: Non-small cell lung cancer; CEF: Cytomegalovirus, Epstein-Barr Virus, influenza virus; GM-CSF: Granulocyte- macrophage colony stimulating factor; IL: Interleukin; IFN: Interferon; TLR: Toll-like receptor; TNF: Tumor necrosis factor; PBMC: Peripheral blood mononuclear cell; BCA: Bicinchoninic acid; HPV: Human papillomavirus.

## Competing interests

H-M. Hu and B.A. Fox are cofounders of UbiVac, which has licensed the autophagosome intellectual property. T.L. Hilton is an employee of UbiVac.

## Authors’ contributions

WY carried out the flow cytometry studies and drafted the manuscript. YX, CP, RV, TLH analyzed data and interpreted results of analysis. TLH contributed reagents for performance of some studies. TM and BAF provided apheresis specimens, BAF and WJU contributed to the critical editing and revision of the manuscript. WZ and HH conceived of the study, and participated in its design and coordination. All authors read and approved the final manuscript.

## Supplementary Material

Additional file 1: Figure S1 pp65 specific CD8^+^ and CD4^+^ T cells response stimulated by DRibbles is dependent on antigen presenting cells. (A) Pure T cells were sorted from human PBMC using Macs Pan T cell isolation kit from Miltenyi Biotec. Then Poly (I:C) and CD40L were added with UbiLT3 pp65 DRibbles or negative control UbiLT3 GFP DRibbles (25ug/ml) to rested PBMC or pure T cells. ICS analysis was done as before. (B) represents CD8^+^ T cells response. (C) represents CD4^+^ T cells response. Click here for file

Additional file 2: Figure S2 CD8^+^ and CD4^+^ T cell responses after stimulated by different TLR agonists. Different agonists were added into PBMCs along with UbiLT3 pp65 DRibbles. (A) CD8^+^ T cell response. (B) CD4^+^ T cell response. Click here for file

Additional file 3: Figure S3 Compare the abilities of GM-CSF/IL4 and Poly (I:C) to enhance T cells response with other cytokines. PBMCs were cultured with cytokines for 12 hours, then HEK 293 pp65 Dribbles were added along with Poly (I:C) or Poly (I:C) and CD40L. (A,B) shows the data that compares GM-CSF + IL-4 with GM-CSF only with or without Poly (I:C) + CD40L. (C,D) shows the data comparing GM-CSF + IL-4 with GM-CSF + IFN-α-2b, IFN-α-2b and GM-CSF + IL-4 + IFN-α-2b. (E,F) DRibbles were collected from HEK 293 T cells that expressed pp65 protein or OVA protein. PBMCs were loaded with 25ug/ml HEK 293 T pp65 DRibbles or control HEK 293 T OVA DRibbles. At the same time, Poly (I:C) was added into the system with or without other cytokines. Then ICS analysis was done as before. Click here for file

Additional file 4: Figure S4 Treatment with bortezomib enhances the abilities of cells and DRibbles to stimulate Ag-specific CD8^+^ T-cell. The UbiLT3 pp65 cell line was cultured with or without bortezomib for 48 hours. DRibbles, cell lysates and whole cells were prepared from bortezomib treated and untreated groups and added to PBMCs as a stimulator. (A,B) shows the CD8^+^ T cell response in donor #1 and #2. (C,D) shows the CD4^+^ T cell response in donor #1 and #2. Click here for file
