# Supplementary figures and images for: Cross-presentation of viral antigens in dribbles leads to efficient activation of virus-specific human memory t cells
Source: J Transl Med. 2014 Apr 16;12:100. doi: 10.1186/1479-5876-12-100 (PMC4021424; doi:10.1186/1479-5876-12-100)

# Supplemental Figure 1.

A

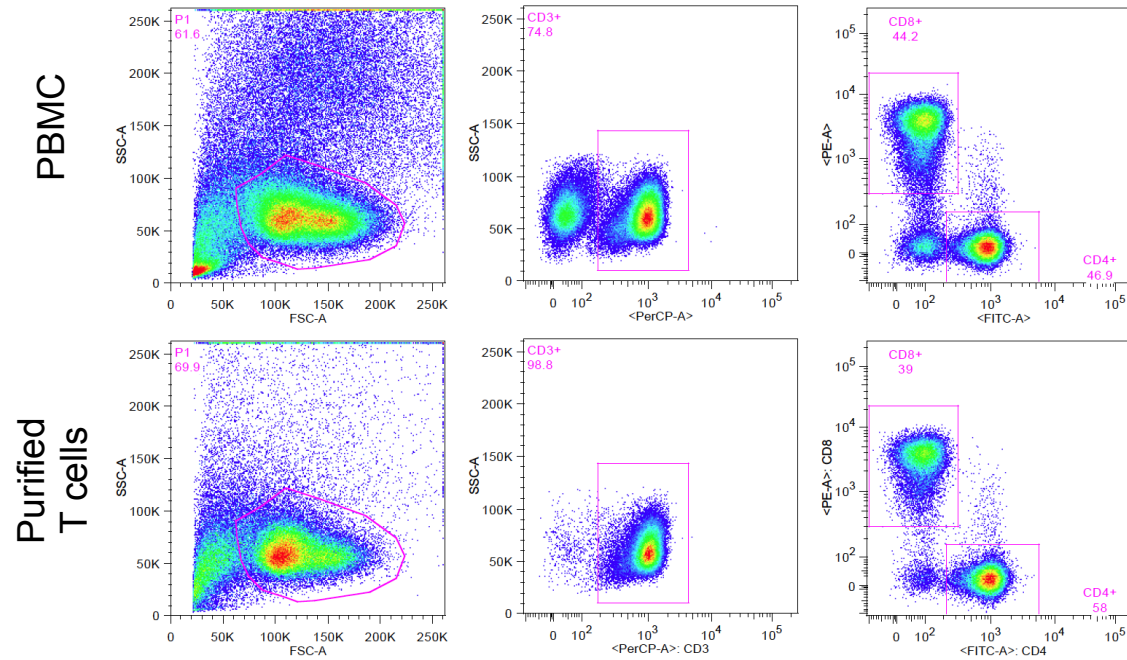

B

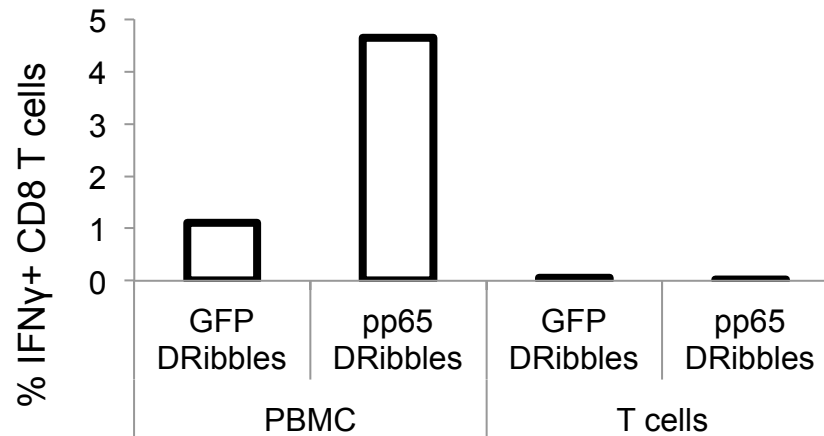

C

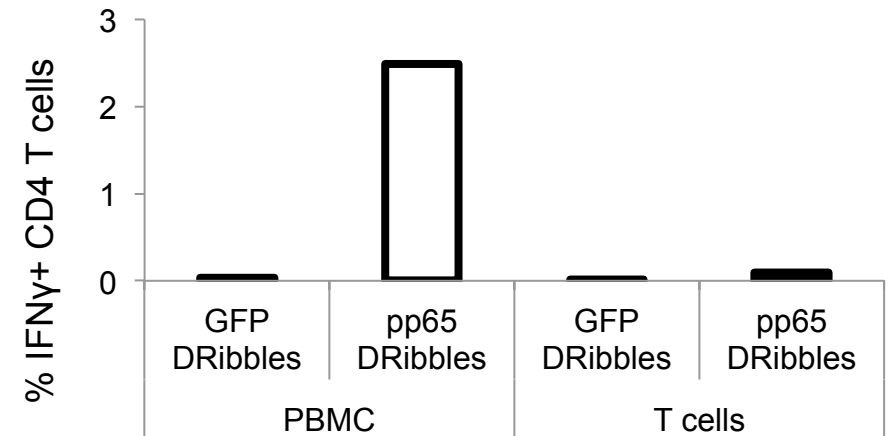

Supplement: Additional file 1: Figure S1 — pp65 specific CD8+ and CD4+ T cells response stimulated by DRibbles is dependent on antigen presenting cells. (A) Pure T cells were sorted from human PBMC using Macs Pan T cell isolation kit from Miltenyi Biotec. Then Poly (I:C) and CD40L were added with UbiLT3 pp65 DRibbles or negative control UbiLT3 GFP DRibbles (25ug/ml) to rested PBMC or pure T cells. ICS analysis was done as before. (B) represents CD8+ T cells response. (C) represents CD4+ T cells response. [file 1479-5876-12-100-S1.pdf]

Supplemental Figure 2.

**A**

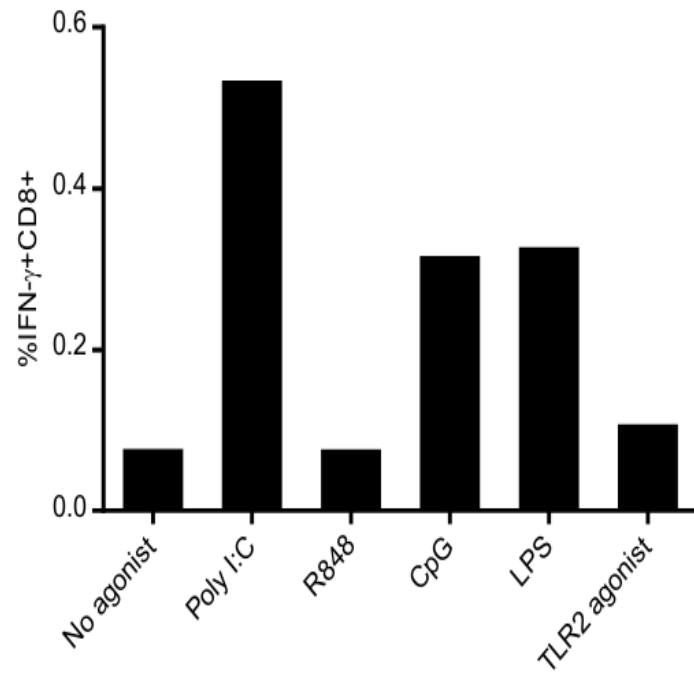

**B**

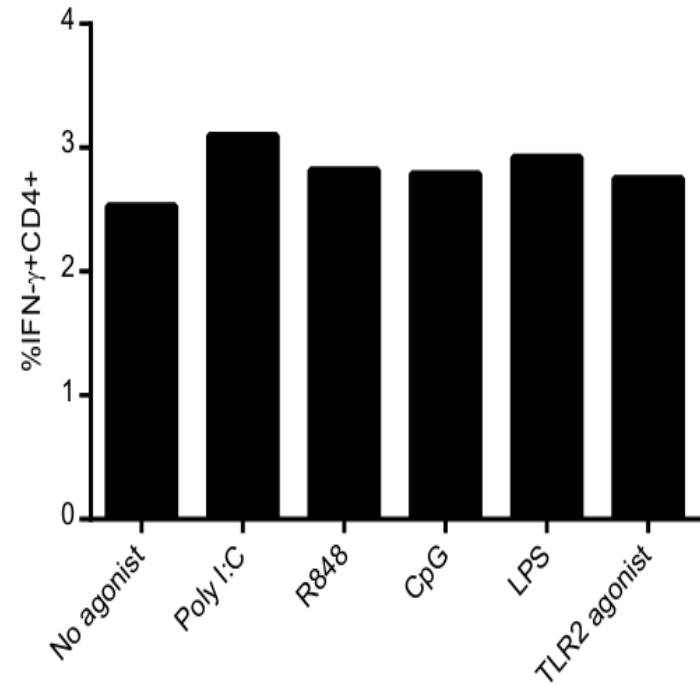

Supplement: Additional file 2: Figure S2 — CD8+ and CD4+ T cell responses after stimulated by different TLR agonists. Different agonists were added into PBMCs along with UbiLT3 pp65 DRibbles. (A) CD8+ T cell response. (B) CD4+ T cell response. [file 1479-5876-12-100-S2.pdf]

Supplemental Figure 3.

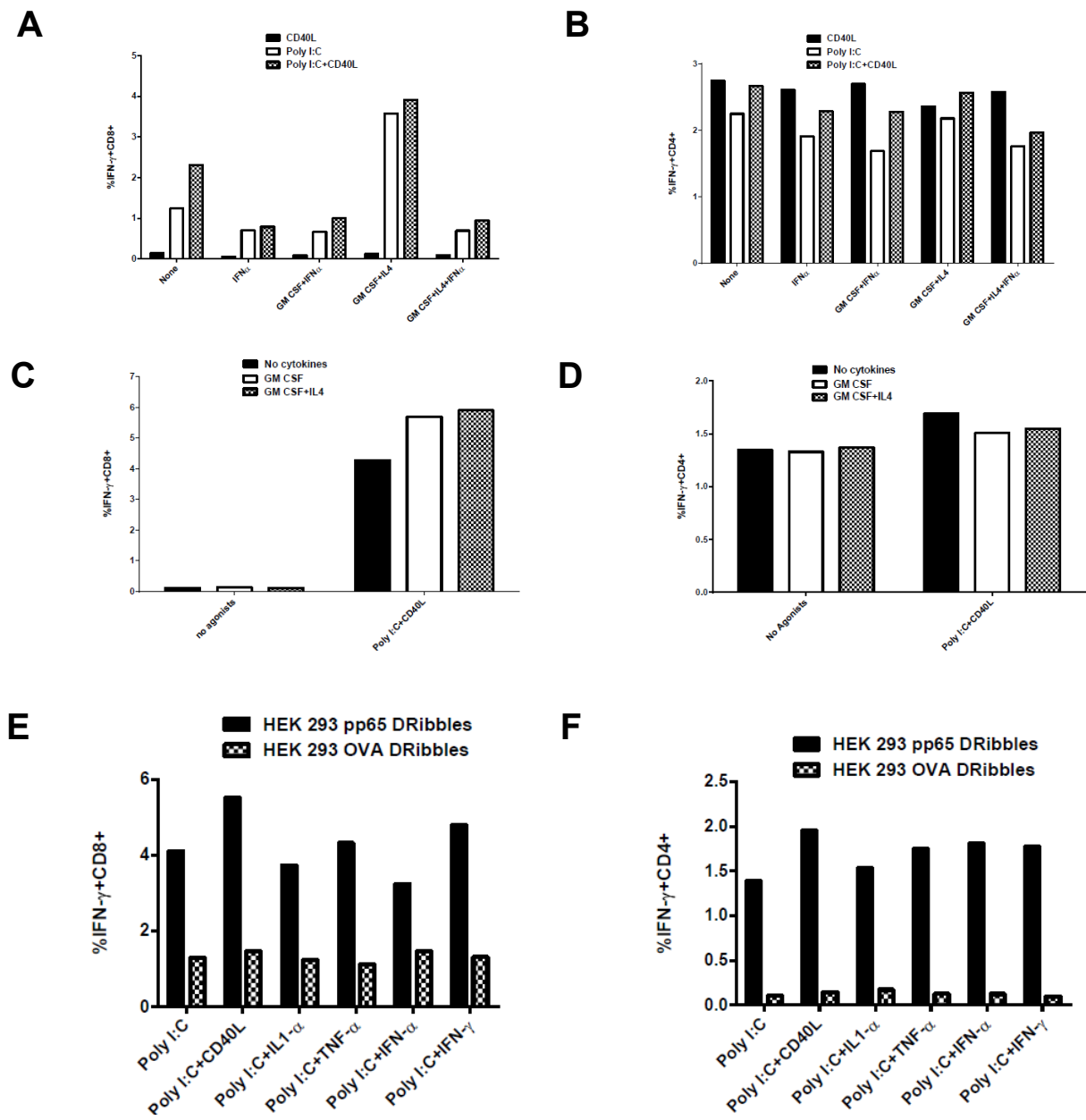

Supplement: Additional file 3: Figure S3 — Compare the abilities of GM-CSF/IL4 and Poly (I:C) to enhance T cells response with other cytokines. PBMCs were cultured with cytokines for 12 hours, then HEK 293 pp65 Dribbles were added along with Poly (I:C) or Poly (I:C) and CD40L. (A,B) shows the data that compares GM-CSF + IL-4 with GM-CSF only with or without Poly (I:C) + CD40L. (C,D) shows the data comparing GM-CSF + IL-4 with GM-CSF + IFN-α-2b, IFN-α-2b and GM-CSF + IL-4 + IFN-α-2b. (E,F) DRibbles were collected from HEK 293 T cells that expressed pp65 protein or OVA protein. PBMCs were loaded with 25ug/ml HEK 293 T pp65 DRibbles or control HEK 293 T OVA DRibbles. At the same time, Poly (I:C) was added into the system with or without other cytokines. Then ICS analysis was done as before. [file 1479-5876-12-100-S3.pdf]

Supplemental Figure 4

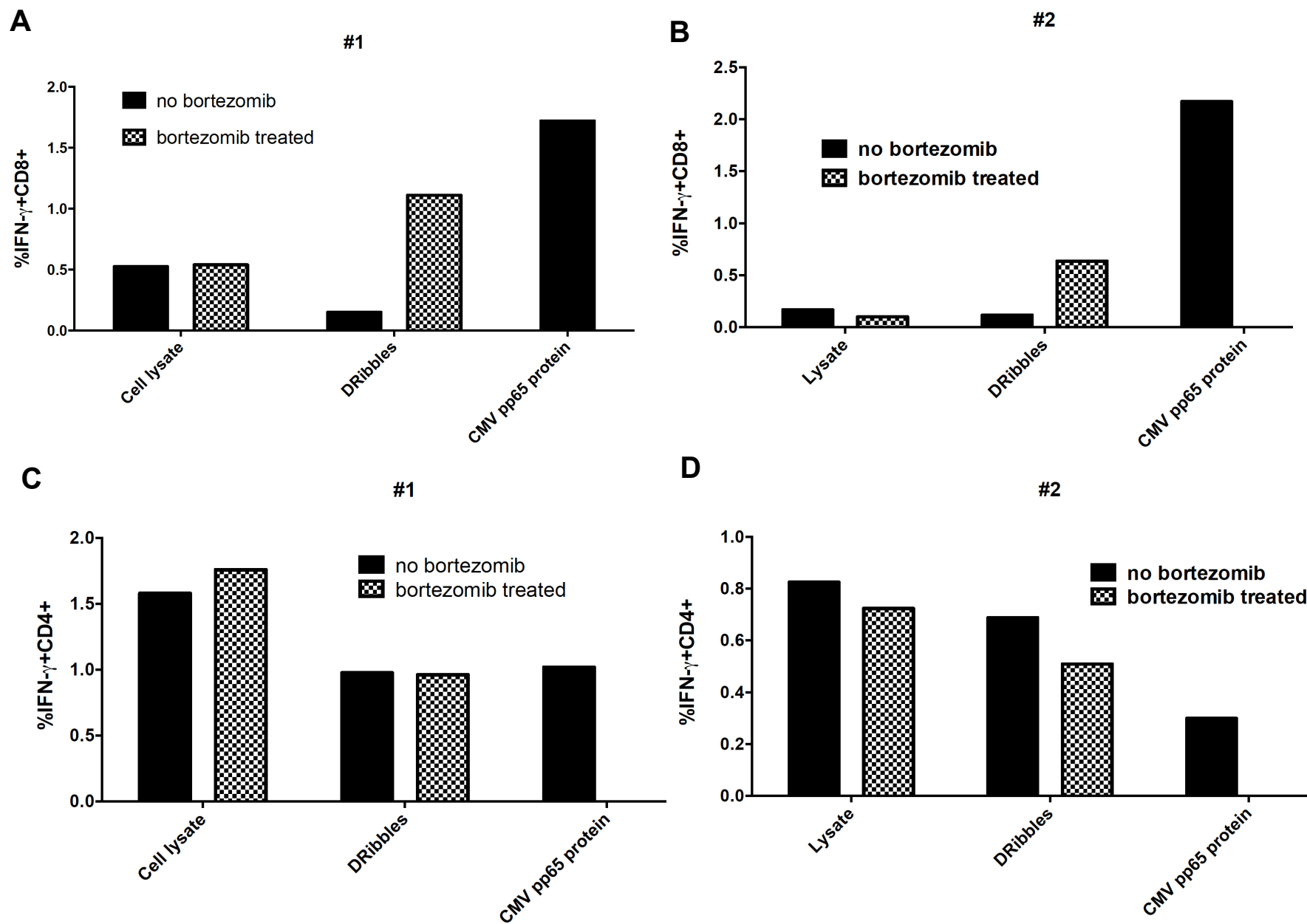

Supplement: Additional file 4: Figure S4 — Treatment with bortezomib enhances the abilities of cells and DRibbles to stimulate Ag-specific CD8+ T-cell. The UbiLT3 pp65 cell line was cultured with or without bortezomib for 48 hours. DRibbles, cell lysates and whole cells were prepared from bortezomib treated and untreated groups and added to PBMCs as a stimulator. (A,B) shows the CD8+ T cell response in donor #1 and #2. (C,D) shows the CD4+ T cell response in donor #1 and #2. [file 1479-5876-12-100-S4.pdf]
